# The Application of Brain-Computer Interface in Upper Limb Dysfunction After Stroke: A Systematic Review and Meta-Analysis of Randomized Controlled Trials

**DOI:** 10.3389/fnhum.2022.798883

**Published:** 2022-03-29

**Authors:** Yang Peng, Jing Wang, Zicai Liu, Lida Zhong, Xin Wen, Pu Wang, Xiaoqian Gong, Huiyu Liu

**Affiliations:** ^1^Department of Rehabilitation Medicine, Yue Bei People’s Hospital, Shaoguan, China; ^2^School of Rehabilitation, Gannan Medical University, Ganzhou, China; ^3^Department of Rehabilitation Medicine, The Seventh Affiliated Hospital, Sun Yat-sen University, Shenzhen, China; ^4^Yue Bei People’s Hospital, Shaoguan, China

**Keywords:** stroke, brain-computer interface, BCI, upper limb dysfunction, meta-analysis

## Abstract

**Objective:**

This study aimed to examine the effectiveness and safety of the Brain-computer interface (BCI) in treatment of upper limb dysfunction after stroke.

**Methods:**

English and Chinese electronic databases were searched up to July 2021. Randomized controlled trials (RCTs) were eligible. The methodological quality was assessed using Cochrane’s risk-of-bias tool. Meta-analysis was performed using RevMan 5.4.

**Results:**

A total of 488 patients from 16 RCTs were included. The results showed that (1) the meta-analysis of BCI-combined treatment on the improvement of the upper limb function showed statistical significance [standardized mean difference (SMD): 0.53, 95% CI: 0.26–0.80, *P* < 0.05]; (2) BCI treatment can improve the abilities of daily living of patients after stroke, and the analysis results are statistically significant (SMD: 1.67, 95% CI: 0.61–2.74, *P* < 0.05); and (3) the BCI-combined therapy was not statistically significant for the analysis of the Modified Ashworth Scale (MAS) (SMD: −0.10, 95% CI: −0.50 to 0.30, *P* = 0.61).

**Conclusion:**

The meta-analysis indicates that the BCI therapy or BCI combined with other therapies such as conventional rehabilitation training and motor imagery training can improve upper limb dysfunction after stroke and enhance the quality of daily life.

## Introduction

In the past two decades, the number of patients with stroke is increasing (Van de Port et al., 2009). Stroke leads to various functional disorders, and the upper limb dysfunction is one of the most common disorders. Upper limb dysfunction not only reduces the quality of patients’ daily life but also makes it difficult to carry out normal rehabilitation training (Feigin et al., 2014). In contrast, it prolongs hospital stay and increases medical costs to patients. Conventional therapies for upper limb dysfunction include postural interventions, functional maneuvers, and exercises. Even though the abovementioned treatments have been widely applied in clinical practice, there is not enough clinical evidence to prove their efficacy. In addition, the single and repeated rehabilitation training are difficult to achieve the expected effect for some patients, and the limited nerve rehabilitation model can no longer meet the increasing rehabilitation needs of patients with stroke (Wang et al., 2010). Therefore, it is very important to find an effective rehabilitation method for stroke.

Brain-computer interface (BCI), an artificial intelligence technology applied in the medical field, is one of the auxiliary means of rehabilitation treatment (Lopez-Larraz et al., 2018). By acting directly on the brain, it can induce the plasticity of the brain and promote the functional reorganization of the brain (Spuler et al., 2018). As we all know, stroke will cause functional or structural changes in the human brain, which will lead to abnormal electroencephalogram (EEG) activity or changes in interhemispheric cerebral excitability (Bolognini et al., 2011; Montenegro et al., 2016). BCI can use brain signals as an alternative channel for communication or equipment control to bypass the brain’s normal output channels of peripheral nerves and muscles. Maybe potentially as a way to influence the neural plasticity process of the brain so as to induce the recovery of normal movement (Daly and Wolpaw, 2008). Therefore, it is becoming a growing area of interest for rehabilitation after stroke (Spuler et al., 2018).

Reviewing previous studies of BCI as treatment of patients with stroke, most clinical research of BCI therapy mainly focused on treatment of upper limb dysfunction after stroke, and several studies reported that BCI has positive findings in clinical. Varkuti et al. (2013) used BCI technology based on motor imagination to treat six patients with chronic stroke and found that five patients were significantly improved in Fugl-Meyer’s upper limb function score. Ang et al.’s (2011) study reported that the application of BCI as treatment of stroke patients can effectively improve the upper limb motor function of patients and can promote the rehabilitation of affected hand and wrist in the sequelae period.

However, the recovery mechanism of BCI on upper limb function after stroke and the effectiveness of BCI treatment are not clear. Besides, the formulation of exercise prescription for BCI treatment may affect treatment effect from many aspects (Rayegani et al., 2014). Therefore, we systematically reviewed the current status of the BCI technology and its application in upper limb dysfunction after stroke. Also, we discussed the efficacy and safety of BCI in treatment of patients after stroke.

## Materials and Methods

### Data Sources and Search Strategy

The following electronic databases that include Chinese and English were searched from their inception up to July 2021. The Chinese databases contained China National Knowledge Infrastructure (CNKI), Wanfang Database, and Chinese Biology Medical disc (CBMdisc). Also, the English databases were as follows: the Cochrane Library, PubMed, EMBASE, and ScienceDirect. At the same time, this meta-analysis also manually searched the references cited in the relevant studies. The research subjects were not limited to Chinese and English. The search terms were as follows: (Brain-computer interface OR Brain-Machine interface OR BCI OR BMI) AND (stroke OR cerebral infarction OR cerebral hemorrhage OR cerebral vascular accident) AND (Randomized controlled trials OR RCTs).

### Inclusion and Exclusion Criteria

We have established strict study criteria for inclusion and exclusion criteria based on study design principles, participants, interventions, and outcomes.

### Types of Study Design Principles

Randomized controlled trials (RCTs) of the effects of BCI on diseases of the central nervous system were first considered. Non-RCT comparative studies were excluded. In addition, case reports, conference papers, and reviews were also excluded. Some incomplete report data, unrelated to research, or unable to convert required data were part of exclusion criteria.

### The Research Participants

Patients clinically diagnosed as stroke was based on the standard diagnostic criteria. There were no restrictions on the age and sex of participants. Participants with significant cognitive, language or psychiatric illnesses, and unwillingness to participate in the experiment were excluded.

### The Interventions

The studies contained an experimental group that was intervened by BCI or the combination of BCI with some conventional treatments, while the control group was treated by routine treatment or blank control or other treatment without BCI.

### The Outcome Measures

Studies that assessed upper limb function such as Fugl-Meyer Assessment for Upper Extremity (FMA-UE) Scale as the primary outcome measures were considered eligible. Other qualities of life and activities of daily living scales were included if they were relevant to the assessment of symptoms.

### Data Extraction

Two authors (YP and JW) independently assessed the titles and abstracts of articles retrieved and evaluated the full-text articles. The data from these articles were validated and extracted according to the following predefined criteria: author, year of publication, characteristics of participants, experimental intervention, control intervention, outcome measures, and adverse events. Any disagreement was resolved by a third party (PW).

### Quality Assessment

To evaluate the quality of included studies, two authors independently assessed the risk of bias using Cochrane’s risk-of-bias tool (Varkuti et al., 2013). Specifically, each domain was judged as belonging to one out of three items: “low risk of bias,” “unclear risk of bias,” and “high risk of bias.”

### Data Analysis

If the eligible outcomes constituted dichotomous data, the relative risk (RR) was calculated, with 95% confidence intervals (CIs). For the clinical effects reported in included studies, the mean change scores and standard deviations (SDs) of the outcomes pre- and post-intervention were extracted for the meta-analyses, as well as the standardized mean difference (SMD), with 95% CIs, was calculated using RevMan 5.4. When there was not enough information about the SDs of the changes, the final values were imputed, as suggested by the Cochrane Handbook for Systematic Reviews of Interventions. The data were then pooled across studies using a random-effects model, with the assumption that each study was not homogeneous. All *P*-values < 0.05 were considered statistically significant. To assess heterogeneity, the chi-square test and Higgins I^2^ statistics were used; an *I*^2^ value of 50% or more was considered an indicator of significant heterogeneity. To determine the robustness of the meta-analysis results with a higher degree of certainty, a sensitivity analysis was implemented. Only studies that had a low risk of bias for random sequence generation and allocation concealment were analyzed.

## Results

### Screening Process and Results of Studies

**FIGURE 1:**
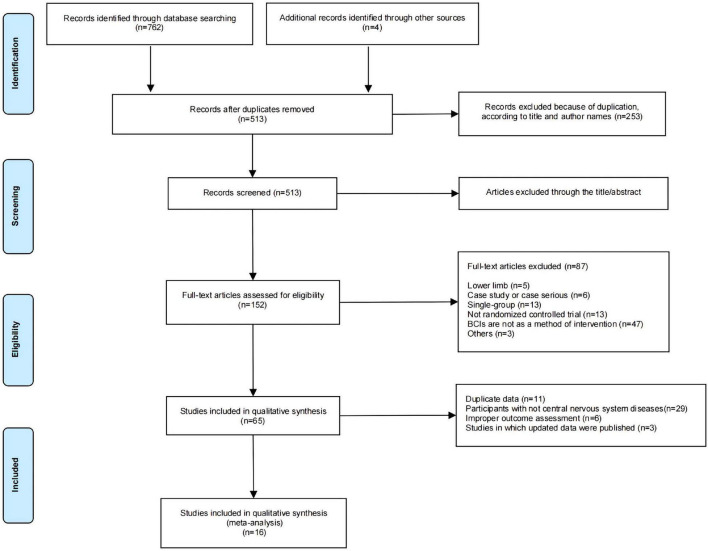
Flowchart of the trial selection process.

A total of 762 potentially relevant studies were identified in the electronic databases. The other four additional studies were identified through other sources. After 253 articles were excluded due to duplication, 513 records were screened according to the title and author names. Based on titles and abstracts, 361 records were excluded because they did not meet the inclusion criteria, and 152 articles were read in full to establish eligibility. Among the full-text articles assessed, 87 were excluded. Ultimately, 65 studies were included in the qualitative synthesis, and only 16 studies achieved sufficient homogeneity in participants, interventions, and outcome measures for inclusion in the quantitative synthesis (Mihara et al., 2013; Li et al., 2014; Ang et al., 2015; Pichiorri et al., 2015; Jang et al., 2016; Kim et al., 2016; Shugeng et al., 2016; Biasiucci et al., 2018; Ying et al., 2018; Ramos-Murguialday et al., 2019; Chen et al., 2020; Hai and Zhiming, 2020; Lee et al., 2020; Sijie et al., 2020; Xianwen et al., 2020; Qiantuo and Tong, 2021). The remaining 49 studies had different outcome measures, improper participants, and duplicate data. Hence, we were unable to perform a meta-analysis on those studies. Figure 1 presents the flowchart of the trial selection process in terms of the Preferred Reporting Items for Systematic Reviews and Meta-Analyses (PRISMA; Cumpston et al., 2019).

### Characteristics of the Included Studies

A summary of included studies is presented in Table 1.

**TABLE 1:** Characteristics of the included clinical trials involving brain-computer interface for the treatment of central nervous system disease.

Study ID	N (E/C)	Age (year)	Disease duration (month)	Intervention
Ramos-Murguialday et al., 2019	16/12	E: 49.3 ± 12.5 C: 50.3 ± 12.2	E: 66.00 ± 45.00 C: 71.00 ± 72.00	E: BCI + con-rehab C: sham BCI + con-rehab
Pichiorri et al., 2015	14/14	E: 64.1 ± 8.4 C: 59.6 ± 12.7	E: 2.70 ± 1.70 C: 2.50 ± 1.20	E: BCI + MI C: MI
Mihara et al., 2013	10/10	E: 56.1 ± 7.9 C: 60.1 ± 8.5	E: 4.90 ± 1.20 C: 4.10 ± 1.30	E: BCI + con-rehab C: sham BCI + con-rehab
Sijie et al., 2020	15/15	E: 50.60 ± 13.46 C: 57.94 ± 8.84	E: 3.00 ± 13.46 C: 2.93 ± 1.44	E: BCI + con-rehab C: sham BCI + con-rehab
Chen et al., 2020	7/7	E: 41.60 ± 12.00 C: 52.00 ± 11.10	E:3.10 ± 1.70 C:3.90 ± 1.50	E: BCI + con-rehab C: sham BCI + con-rehab
Lee et al., 2020	13/13	E: 55.15 ± 11.57 C: 58.30 ± 9.19	E: 7.46 ± 1.61 C: 8.30 ± 1.97	E: BCI + con-rehab C: sham BCI + con-rehab
Ying et al., 2018	16/16	E: 72.43 ± 8.56 C: 76.81 ± 9.57	E: N/A C: N/A	E: BCI + con-rehab C: sham BCI + con-rehab
Jang et al., 2016	10/10	E: 61.10 ± 13.77 C: 61.70 ± 12.09	E: 4.40 ± 0.97 C: 4.10 ± 0.74	E: BCI-FES + con-rehab C: FES + con-rehab
Biasiucci et al., 2018	14/13	E: 56.40 ± 9.90 C: 59.00 ± 12.40	E: 39.80 ± 45.9 C: 33.50 ± 30.5	E: BCI-FES C: sham BCI
Ang et al., 2015	11/14	E: 48.5 ± 13.5 C: 53.60 ± 9.50	E: 12.35 ± 9.38 C: 7.57 ± 5.93	E: BCI-Manus robot C: Manus robot
Li et al., 2014	7/7	E: 66.30 ± 4.90 C: 67.10 ± 6.30	E: 2.20 ± 1.80 C: 2.80 ± 2.00	E: BCI-FES + con-rehab C: FES + con-rehab
Kim et al., 2016	15/15	E: 59.07 ± 8.07 C: 59.93 ± 9.79	E: 8.27 ± 1.98 C: 7.80 ± 1.78	E: BCI-FES + con-rehab C: FES + con-rehab
Shugeng et al., 2016	3/3	E: 48.00 ± 11.26 C: 51.00 ± 14.00	E: N/A C: N/A	E: BCI + MI C: MI
Hai and Zhiming, 2020	30/30	E: 41.77 ± 8.65 C: 40.7 ± 8.15	E: 1.06 ± 0.16 C: 1.09 ± 0.12	E: BCI + con-rehab C: con-rehab
Xianwen et al., 2020	47/47	E: 58.60 ± 2.70 C: 60.20 ± 1.90	E: 0.61 ± 0.06 C: 0.64 ± 0.07	E: BCI + con-rehab C: con-rehab
Qiantuo and Tong, 2021	17/17	E: 49.93 ± 8.82 C: 51.27 ± 8.56	E: 51.53 ± 15.43 C: 51.87 ± 14.87	E: BCI-FES C: FES

**Study ID**		**Outcome measures**	**Duration**	**Adverse effects**

Ramos-Murguialday et al., 2019		FMA-UE GAS MAL MAS	5 times a week for 4 weeks	N/A
Pichiorri et al., 2015		FMA-UE MRC MAS	3 times a week for 4 weeks	N/A
Mihara et al., 2013		FMA-UE ARAT MAL	3 times a week for 2 weeks	N/A
Sijie et al., 2020		FMA-UE MBI MAS	5 times a week for 4 weeks	N/A
Chen et al., 2020		FMA-UE WMFT MBI MAL	3 times a week for 4 weeks	N/A
Lee et al., 2020		FMA-UE WMFT MBI MAL	3 times a week for 4 weeks	N/A
Ying et al., 2018		FMA-UE MBI	3 times a week for 8 weeks	N/A
Jang et al., 2016		MAS	5 times a week for 6 weeks	N/A
Biasiucci et al., 2018		FMA-UE MRC MAS	2 times a week for 5 weeks	N/A
Ang et al., 2015		FMA-UE	3 times a week for 4 weeks	Hemiplegic shoulder pain
Li et al., 2014		FMA-UE ARAT	3 times a week for 8 weeks	Allergic to electrode pads
Kim et al., 2016		FMA-UE MAL MBI ROM	5 times a week for 4 weeks	N/A
Shugeng et al., 2016		MAS FMA	3 times a week for 4 weeks	N/A
Hai and Zhiming, 2020		FMA-UE MBI WMFT	5 times a week for 4 weeks	N/A
Xianwen et al., 2020		FMA-UE MBI	2 weeks was one course resting 3 days for 7 weeks	N/A
Qiantuo and Tong, 2021		FMA-UE MBI	5 times a week for 4 weeks	N/A

*E, experience group; C, control group; BCI, brain-computer interface; MI, motor imagery; FES, functional electrical stimulation; FMA-UE, Fugl-Meyer Assessment for Upper Extremity; GAS, Goal Attainment Scale; MAL, motor activity log; MAS, Modified Ashworth Scale; MRC, Medical Research Council Scale; ARAT, Action Research Arm Test; MBI, Modified Barthel Index; WMFT, Wolf Motor Function Test; ROM, range of motion.*

### Risk of Bias

**FIGURE 2:**
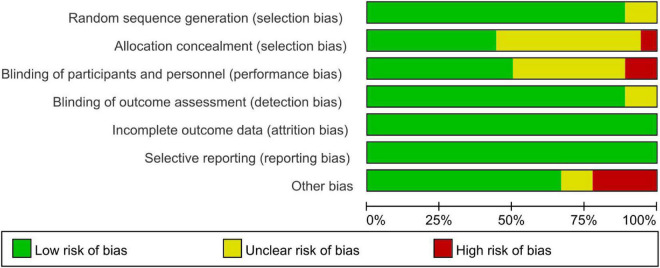
Graph of the risk of bias: percentage across all included studies.

**FIGURE 3:**
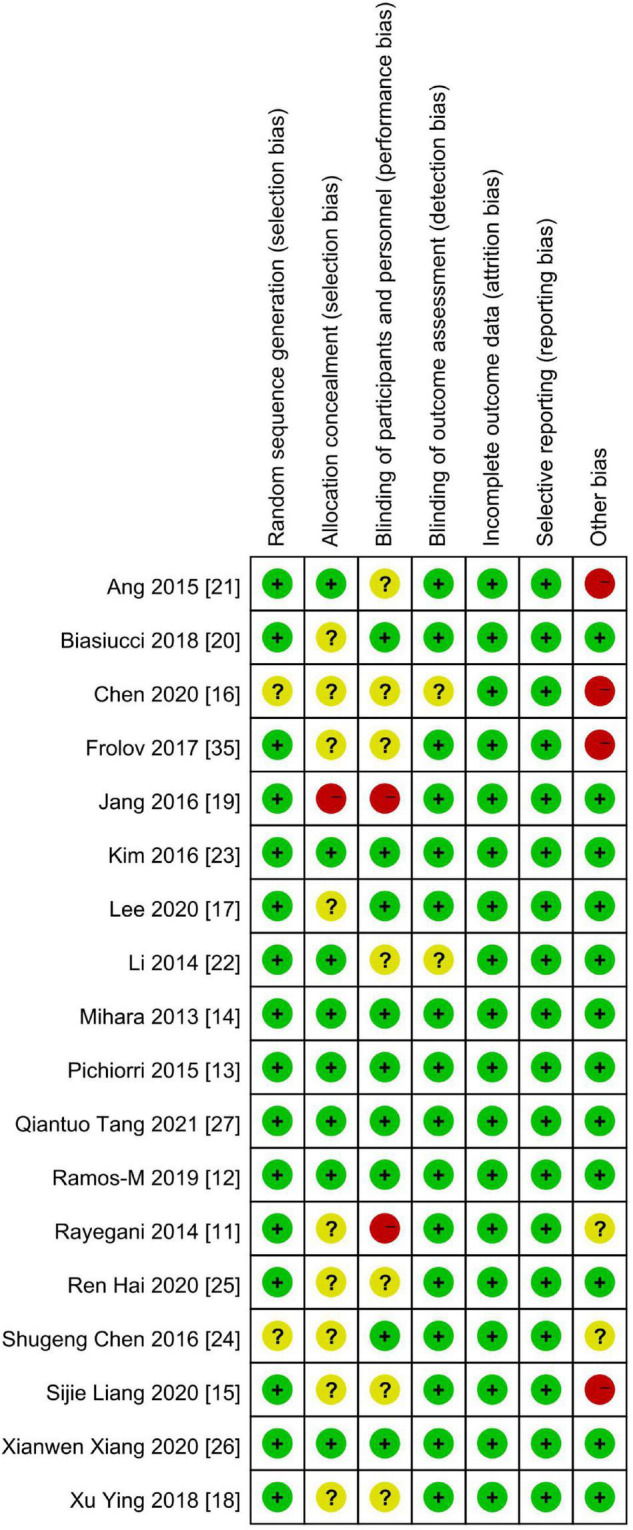
Summary of the risk of bias: review authors’ judgments about each risk-of-bias item in each included study. “+,” low risk of bias; “?,” unclear risk of bias; “–,” high risk of bias.

Figures 2, 3 demonstrate the risk of bias in the included studies.

### Meta-Analysis

In this study, all forest plots evaluated immediate effects after treatment.

### Fugl-Meyer Assessment Score

**FIGURE 4:**
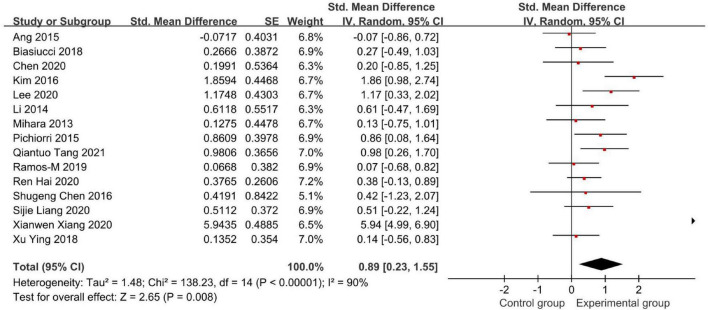
Forest plot of the Fugl-Meyer Assessment (FMA) score to evaluate the effect of brain-computer interface (BCI) on upper limb dysfunction after stroke.

**FIGURE 5:**
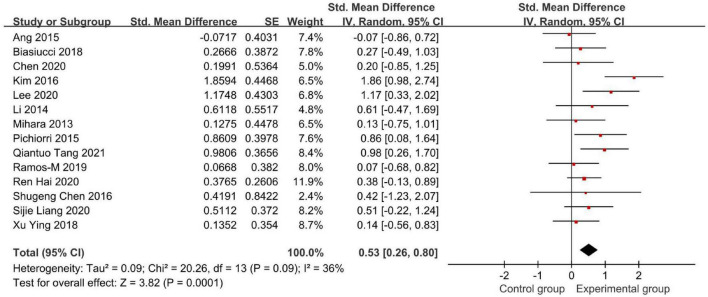
Forest plot of the FMA score after omitting Xianwen Xiang’s study to evaluate the effect of BCI on upper limb dysfunction after stroke.

Figures 4, 5 demonstrate the Fugl-Meyer Assessment (FMA) score.

Fifteen studies used the FMA to assess the effectiveness of BCI as an intervention to upper limb dysfunction after stroke (Figure 4). We found an SMD of 0.89 (95% CI: 0.23–1.55), meaning that the average FMA-UE score of the experimental group is separated by 89% of the pooled SD from the control group. But the result had a high heterogeneity (*I*^2^ = 90%).

Therefore, we conducted a sensitivity analysis by omitting one study in each term to assess the influence of individual studies on the pooled result. Finally, we removed Xianwen Xiang’s studies, which have a great impact on heterogeneity, and recalculated the combined estimate on the remaining studies (Figure 5). As a result, we demonstrated that BCI treatment rather than the control group had a significantly greater effect on upper limb recovery after stroke in the FMA-UE score (SMD: 0.53, 95% CI: 0.26–0.80, *P* = 0.0001). Also, the heterogeneity was not significant (*I*^2^ = 36%, *P* = 0.09).

### Modified Barthel Index Score

**FIGURE 6:**
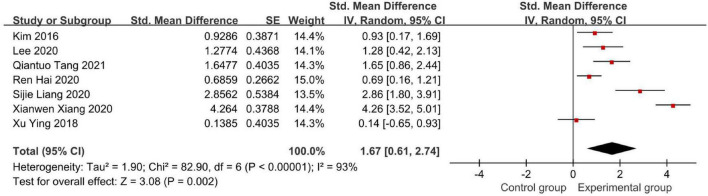
Forest plot of the Modified Barthel Index (MBI) score to evaluate the effect of BCI on upper limb dysfunction after stroke.

Figure 6 demonstrates the Modified Barthel Index (MBI) score.

In seven studies that used the MBI scale, our meta-analysis showed that SMD = 1.67 with 95% CI: 0.61–2.74, *I*^2^ = 93% (Figure 6). Similarly, we conducted a sensitivity analysis, in which 1 study was removed at each time, which was performed to evaluate the stability of the results. But there was no apparent fluctuation. This analysis confirmed the stability of the results. According to the actual MBI scores in these studies, both experimental groups and control groups showed improvement after intervention in these seven studies. Furthermore, the experimental groups recovered more in terms of the MBI scale than the control groups.

### Modified Ashworth Scale Score

**FIGURE 7:**
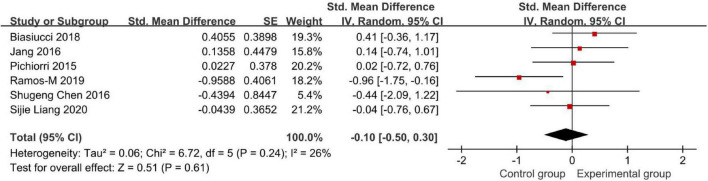
Modified Ashworth Scale (MAS) to evaluate the effect of BCI on upper limb dysfunction after stroke.

Figure 7 demonstrates the Modified Ashworth Scale (MAS) score.

Six studies compared the effects of BCI treatment using the MAS score (Figure 7). The meta-analysis showed that the BCI intervention was not significantly superior to conventional treatment on the amount of use of their affected hand (SMD: −0.10, 95% CI: −0.50 to 0.30, *P* = 0.61). The heterogeneity was not significant (*I*^2^ = 26%). Regarding actual MAS scores, Pichiorri et al. (2015), Biasiucci et al. (2018), and Ramos-Murguialday et al. (2019) studies found an increase in MAS (i.e., increase in spasticity). Also, the last three studies showed a decrease in the MAS scores. Among these six studies, only Shugeng et al. (2016), Ramos-Murguialday et al. (2019), and Sijie et al. (2020) results showed that the BCI intervention was superior to conventional treatments. However, the analysis results are not conclusive due to the low number of studies. Therefore, it needs to be discussed and analyzed carefully.

## Discussion

This study aims to analyze the effectiveness of BCI in improving upper limb function after stroke. This updated meta-analysis included a total of 16 studies, which also included 488 patients. All the included studies showed that BCI played a positive role in promoting the upper limb rehabilitation of patients after stroke. In this study, only Li et al. (2014) indicated that subjects were allergic to electrode pads. Ang et al. (2015) indicated one case of shoulder joint pain. In addition, most studies did not mention adverse reactions that occurred.

Bai et al. (2020) conducted the meta-analysis investigating the immediate effect as well as the long-term effect of BCI and observed a positive and significant benefit of BCI with 18 single-group studies and 15 controlled studies. Another meta-analysis published by Kruse et al. (2020) indicated that BCI training enhanced brain function recovery and upper limb function after stroke by analyzing 14 RCTs. A total of 14 RCTs were included in these 2 meta-analyses. With 2 additional RCTs being included, our study updated the databases of the trial, and our study was able to investigate the specific performance of upper limb function in common scales such as FMA, MBI, and MAS after intervention.

The BCI repairs functional control connections between external limbs and the brain by rebuilding the cortex of the damaged brain. The process of neural recovery is an important basis for a variety of functions, and certain neuropathological changes are leading to different dysfunctions in many nervous system diseases. BCI has been verified to have a positive effect based on the functional mechanisms such as cortical excitability (Moher et al., 2009), cerebral plasticity, and functional connectivity (Daly and Wolpaw, 2008; Sawaki et al., 2014; Shimamura et al., 2017). Therefore, BCI is transforming to a rehabilitation method of upper limb dysfunction based on the principle of neuroplasticity.

The FMA of the upper limb included eight subitems, namely, reflex, flexor cooperative movement, extensor cooperative movement, activity with the cooperative movement, activity out of the cooperative movement, normal reflex, wrist joint stability, and hand movement and coordination ability and speed. The difference between the experimental group and the control group was statistically significant (*P* < 0.05) when the FMA score was used as the outcome measure in our study. Ang et al.’s (2015) study showed that the performance of the BCI group was slightly lower than that of the control group. But only 13% of repetitions in the BCI group compared with those in the control group, which may lead to this result. Therefore, we can conclude that safe and effective BCI intervention can improve the functional performance of upper limb dysfunction after stroke. However, one of the most significant difficulties during rehabilitation after stroke is the recovery of fine upper limb skills (Jacquin-Courtois, 2015). Shugeng et al.’s (2016) experiment showed that the functional improvement of patients was mainly reflected in the hand after BCI therapy. Besides, Mihara et al. (2013) performed the neurofeedback only affected in the distal part of the affected arm. However, Ang et al.’s (2015) and Kim et al.’s (2016) studies showed that the rehabilitation led to positive effects on shoulder and elbow. From another perspective, Mihara et al. (2013), Li et al. (2014), and Frolov et al. (2017) found that there was no significant effect on the Action Research Arm Test (ARAT) scores between BCI groups and control groups after intervention. However, Mihara et al. (2013) found that the cortical activation change was correlated with the recovery of the hand function, suggesting that the modulation of the excitability in the BCI and networks augments the functional recovery. Consistent with our findings, we cannot clarify that BCI has a better effect on improving the wrist in the upper limb after stroke, but improving the function of the whole upper limb is explicit. In addition, Ang et al. (2015) found that it is a greater improvement in the FMA scores at 4 weeks of BCI intervention during treatment and the follow-up time. Improvement in the FMA scores following BCI intervention was also demonstrated in Mane et al.’s (2019) study, which showed that the FMA scores improved better at the follow-up of 4 weeks. In most of the included experiments, the intervention time of BCI was mostly 4 weeks. The efficacy of a 4-week intervention with BCI was also demonstrated in the profile.

The ultimate goal of rehabilitation is to improve the patient’s quality of life and to return to society. The MBI scores (SMD = 1.67) were evaluated in seven studies. The results of the analysis showed that the BCI intervention in treatment of poststroke upper limb dysfunction has improved the quality of life of patients and their ability to take care of themselves at the same time, which was consistent with Lin’s finding (Lin et al., 2021).

## Limitations

This meta-analysis may have some heterogeneity in methodological quality. It is because some studies did not mention the details of these patients with stroke, such as stroke location and so on. There are also some differences in the outcome indicators of each study. In addition, to study the best treatment of BCI in improving dysfunction, it is necessary to expand the sample size and conduct the long-term follow-up for further analysis to determine the clinical value of BCI.

## Conclusion

Our updated meta-analysis with 16 RCTs indicated that BCI therapy or combined with other therapies such as routine rehabilitation training had favorable effects on upper limb function recovery after stroke. These findings also suggest that intervention duration for 4 weeks had the largest effect size. We also observed that BCI is a safe neuromodulator intervention to improve upper limb functions in patients with stroke.

## Data Availability Statement

The original contributions presented in the study are included in the article/supplementary material, further inquiries can be directed to the corresponding authors.

## Author Contributions

HL and PW designed the study. YP and JW negotiated article methodology and executed search strategy. XW managed the search results of the database. YP and ZL performed the data analysis. LZ and XG provided suggestions for the revision of the manuscript. All authors contributed to screening procedures, data curation, and additional supplementary material.

## Conflict of Interest

The authors declare that the research was conducted in the absence of any commercial or financial relationships that could be construed as a potential conflict of interest.

## Publisher’s Note

All claims expressed in this article are solely those of the authors and do not necessarily represent those of their affiliated organizations, or those of the publisher, the editors and the reviewers. Any product that may be evaluated in this article, or claim that may be made by its manufacturer, is not guaranteed or endorsed by the publisher.
